# Myelofibrosis-Related Anemia: Current and Emerging Therapeutic Strategies

**DOI:** 10.1097/HS9.0000000000000001

**Published:** 2017-12-20

**Authors:** Leonard Naymagon, John Mascarenhas

**Affiliations:** Tisch Cancer Institute, Icahn School of Medicine at Mount Sinai, New York, NY

**Keywords:** anemia, anti-fibrotic agents, myelofibrosis, novel JAK inhibitors, TGF-β ligand traps

## Abstract

Myelofibrosis (MF) is a clonal hematopoietic stem cell disorder characterized by pathological myeloproliferation and aberrant cytokine production resulting in progressive fibrosis, inflammation, and functional compromise of the bone marrow niche. Patients with MF develop splenomegaly (due to extramedullary hematopoiesis), hypercatabolic symptoms (due to overexpression of inflammatory cytokines), and anemia (due to bone marrow failure and splenic sequestration). MF remains curable only with allogeneic hematopoietic stem cell transplantation (ASCT), a therapy that few MF patients are deemed fit to undergo. The goals of treatment are thus often palliative. The approval of the JAK inhibitor ruxolitinib has done much to address the burden of splenomegaly and constitutional symptoms of patients with MF; however, therapy-related anemia is often an anticipated downside. Anemia thus remains a challenge in the management of MF and represents a major unmet need. Intractable anemia depresses quality of life, portends poor outcomes, and can act to restrict access to palliative JAK inhibition in some patients. While therapies for MF-related anemia do exist, they are limited in their efficacy, durability, and tolerability. Therapies currently in development promise improved anemia-specific outcomes; however, are still early in the pathway to regulatory approval and regular clinical use. In this review, we will discuss established and emerging treatments for MF-related anemia. We will give particular attention to developmental therapies which herald significant progress in the understanding and management of MF-related anemia.

## Introduction

Myelofibrosis (MF) is a clonal hematopoietic stem cell disorder characterized by pathological bone marrow myeloproliferation.^1^ MF may occur de novo (primary myelofibrosis) or arise from a preexisting myeloproliferative neoplasm, namely polycythemia vera or essential thrombocytosis. MF is defined by progressive bone marrow fibrosis, the result of a nonclonal fibroblastic response to inflammatory and fibrogenic cytokines produced by aberrant clonal myeloid cells, most prominently megakaryocytes.^2^ This disruption of the medullary erythropoietic niche is the primary mechanism governing the bone marrow failure and anemia which typify MF. MF remains curable only after allogeneic hematopoietic stem cell transplantation (ASCT), a therapy which is particularly challenging for MF patients due to advanced age, competing comorbidities, and lack of viable donor options.^3^ As such, medicinal treatments are largely palliative and directed toward amelioration of disease sequelae, such as splenomegaly, hypercatabolic symptoms, and anemia.^4^ While the emergence of JAK2 inhibition has provided substantial benefit for splenomegaly and systemic symptoms, anemia and thrombocytopenia have remained challenges in the management of MF and are glaring unmet needs.^5^ Existing approaches including transfusion, erythropoiesis-stimulating agents (ESAs), androgens, corticosteroids, immunomodulators, and splenectomy produce inconsistent results and are fraught with complications.^6^ Emerging therapies including novel JAK2 inhibitors, anti-fibrotic agents, and TGF-β ligand traps spark significant optimism in improving MF-related cytopenias, however, remain unproven.^7^ In this review we will discuss the clinical significance and pathogenesis of anemia in MF and explore both established and emerging treatments. In particular, we will focus on novel therapies currently in development, which portend a forthcoming transformation in the management of MF-related anemia.

## The significance of anemia in myelofibrosis

Anemia is among the cardinal features of MF. Nearly 40% of MF patients have hemoglobin (Hb) levels <10 g/dL at diagnosis, and nearly one-quarter are already RBC transfusion-dependent.^8^ Virtually all patients with MF will eventually develop anemia. Anemia has consistently been associated with inferior quality-of-life measures among MF patients and response to anemia-targeted therapies has been associated with improvement in quality of life.^9^ With the progress in treatment of splenomegaly and hypercatabolic symptoms in the era of JAK2 inhibition, anemia is left as the major negative determinant of MF-related quality of life. Furthermore, anemia is the disease feature most consistently associated with poor prognosis in MF.^10^ Hemoglobin <10 g/dL is an integral component of the Dynamic Prognostic Scoring System (DIPSS) and DIPSS-Plus scores for estimating prognosis in MF, with transfusion-dependency included as an additional adverse feature in the DIPSS-Plus model.^11^

## The pathogenesis of anemia in myelofibrosis

**Figure 1:**
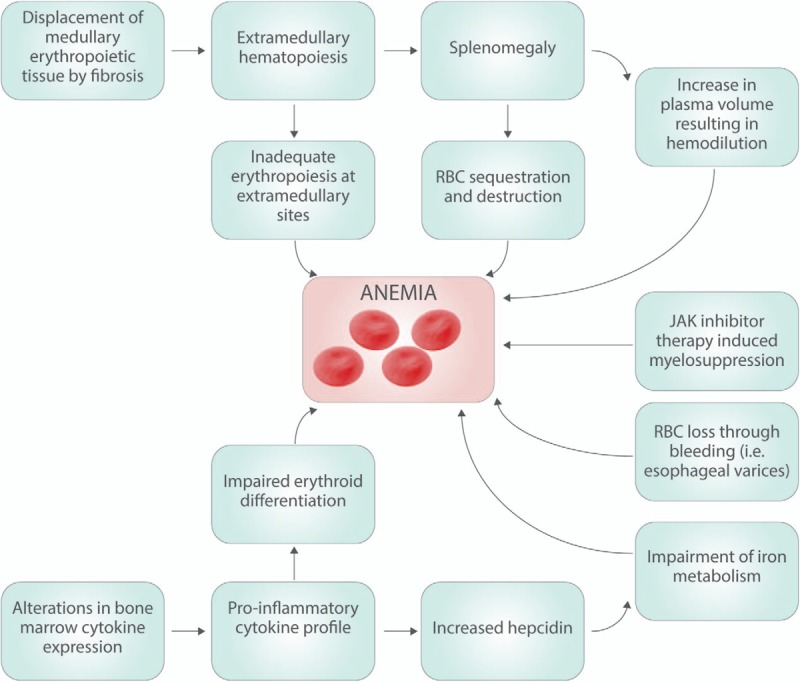
**The pathogenesis of anemia in myeolofibrosis is the result of a multifactorial process, which is only partially understood.** The relative contributions of each of the above etiologies vary from patient to patient, and this variability in pathogenesis may explain the variability in responses to different therepeutic modalities. RBC = red blood cell.

Anemia in MF is the result of a multifactorial process, which is incompletely understood.^12^ The displacement of medullary erythropoietic tissue by fibrotic stroma has long been regarded as the central pathogenic process, and although it largely retains this position today, it is no longer considered the only contributing etiology.^6^ As exiled erythropoietic tissue migrates to the spleen and other extramedullary sites it is met with suboptimal milieus for erythrogenesis and RBC maturation, leading to ineffective erythropoiesis, and preventing extramedullary sites from adequately compensating for the loss of productive marrow.^13^ The splenomegaly induced by extramedullary erythropoiesis prompts sequestration and destruction of circulating RBCs thus exacerbating anemia.^14^ Furthermore, plasma volume increases with spleen size often leading to a component of dilutional anemia.^6^ In addition to progressive fibrosis, the bone marrow niche in MF is characterized by abnormal cytokine expression, which promotes both local and systemic inflammation.^15^ The resulting proinflammatory environment disrupts erythrogenesis in any residual functioning marrow. Upregulation of inflammatory cytokines in the bone marrow of MF patients has also been associated with upregulation of circulating hepcidin, which interferes with iron metabolism and utilization in a manner not unlike that of anemia of chronic disease.^16^ A component of anemia in MF may sometimes be therapy related. Ruxolitinib (Jakafi, Incyte Corporation, Wilmington, Delaware, United States), the only JAK inhibitor approved for intermediate- and high-risk MF, may cause therapy related anemia via suppression of residual marrow function.^17,18^ MF patients harboring *CALR* or *MPL* mutations are less likely to develop anemia compared to triple negative patients (*CALR, MPL*, and *JAK2* wild type).^18,19^ The mechanisms behind these clinical differences remain unknown and are the subject of ongoing investigation. Rare contributors to anemia among some patients may also include autoimmune hemolysis or bleeding due to severe thrombocytopenia.^20^ Certainly patients with MF can concomitantly have any of the common causes of anemia such as iron, B12, or folate deficiency, chronic blood loss, hemolysis or inflammation from unrelated comorbidities, and these should always be clinically evaluated and addressed accordingly (Figure 1).

## Established treatments

### Transfusion

As a testament to the poor efficacy of established treatments for MF-related anemia, RBC transfusion-dependence remains an often inescapable hallmark of late stage disease. Per the International Working Group-Myeloproliferative Neoplasms Research and Treatment (IWG-MRT) and European LeukemiaNet (ELN) consensus report, transfusion-dependence is defined as transfusion of >6 units of RBC in a 12-week period for an Hb level of <8.5 g/dL, in the absence of bleeding or treatment-induced anemia.^21^ Nearly one-quarter of MF patients are RBC transfusion-dependent at time of diagnosis and nearly all will eventually develop RBC transfusion-dependence.^8^ RBC transfusion-dependence is an independent predictor of poor prognosis and is inversely correlated with quality of life.^9,11,22,23^ Rather than representing a true therapy, frequent RBC transfusion signals the failure and exhaustion of other available treatments, and heralds end-stage disease. Complications of chronic RBC transfusion-dependence include alloimmunization and iron overload; however, patients often die before the accumulation of alloantibodies and tissue iron stores become extensive.^24^ Given the short survival associated with transfusion-dependence, iron chelation is typically not pursued unless the patient is deemed an ASCT candidate.^23,25^ Case reports exist of improvement in anemia with chelation; however, there are no studies, either prospective or retrospective, to validate these sporadic observations.^26–29^ There were retrospective data to support chelation in transfusion-dependent patients with low-risk myelodysplastic syndromes (MDS); however, these findings cannot yet be extrapolated to MF.^29^

### Erythropoiesis stimulating agents

Although studies have been small, there is evidence for the effectiveness of ESAs among transfusion-independent MF patients with low serum erythropoietin (S-Epo).^30^ Cervantes et al administered recombinant human erythropoietin (rhEPO) at an initial dose of 10,000 U thrice per week to 20 patients with MF and anemia.^31^ Four patients (20%) achieved complete response (CR) defined as RBC transfusion cessation with a normal Hb level for at least 8 weeks, and an additional 5 patients (25%) achieved partial response (PR) defined as a 50% decrease in RBC transfusional requirements to maintain an Hb of at least 10 g/dL. Pretreatment factors associated with response included baseline RBC transfusion-independence and low S-Epo (<125 U/L). The investigators repeated this trial with darbepoetin-alpha, again enrolling 20 MF patients with anemia, and demonstrated a CR rate of 30% with an additional 10% of patients attaining PR.^32^ None of the patients with high S-Epo levels achieved response. In both trials, less than half of responding patients were able to maintain response through a median follow-up of 12 months. A similar study by Tsiara et al demonstrated comparable results and confirmed that patients with low S-Epo levels were more likely to respond.^33^ A more recent retrospective study by Huang et al which included 43 MF patients treated at a single institution demonstrated an OR (defined as a minimum 2.0 g/dL increase in Hb level or transfusion-independence for a minimum of 1-month) of only 23%.^34^ None of the transfusion-dependent patients demonstrated response. Based on the findings of these studies, ESAs should only be used in those MF patients who are RBC transfusion-independent and have low S-Epo levels (<125 U/L). However, even among candidate patients, less than half will respond, and less than half of responders can be expected to maintain response for over a year. Nonresponders at 3 months are unlikely to benefit and should discontinue therapy. Treatment seems well tolerated with no reports of thrombosis in the above studies.

### Androgens

Small prospective studies of the androgens oxymetholone, fluoxymesterone, nandrolone, and testosterone, conducted in the 1980s established the effectiveness of androgen therapy in MF-related anemia with response rates ranging from 30% to 60% (although response criteria were less stringent than those in contemporary trials).^35–37^ Danazol, a synthetic attenuated androgen, is preferred over the above agents given its superior safety and tolerability and comparable efficacy. Cervantes et al demonstrated a 37% response rate to danazol among 30 MF patients in 2005, although the study was limited by lenient response criteria and short follow-up.^38^ This same group of investigators repeated the trial in 2015 with a larger cohort (50 patients), evaluated on more stringent and standardized response requirements (defined by the IWG-MRT/ELN criteria), with longer median follow-up (36 months).^39^ Response was achieved in 30% of patients, including 44% of transfusion-independent patients and 19% of transfusion-dependent patients. Median time to response was 5 months and median duration of response was 14 months. The most frequent toxicity was grade 1 to 2 transaminitis, which occurred in 16% of patients and improved with dose reduction, although 2 patients did develop severe cholestatic hepatitis, which resolved after drug discontinuation. Due to concern regarding androgenic promotion of prostate cancer, all enrolled patients were monitored via prostate-specific antigen (PSA) testing, with a single patient eventually discontinuing therapy due to emergent prostate cancer while on study. Danazol was initially dosed at 600 mg daily and continued for at least 6 months or until response was achieved, at which point the dose was titrated to the minimum necessary to maintain response. Although it has been shown to elicit responses in less than half of RBC transfusion-independent MF patients, and less than a quarter of RBC transfusion-dependent patients, its tolerability and relatively benign safety profile make danazol a reasonable option for MF patients with symptomatic anemia. Patients with concomitant thrombocytopenia may derive added benefit as danazol may also improve platelet counts in some patients. Nevertheless, pretreatment expectations should be moderated and liver function and PSA should be monitored.

### Corticosteroids

Prednisone has demonstrated similar response rates to danazol, even though with more limited trial data, and at the cost of greater toxicity.^6^ Its mechanism of action in this setting is not understood, though it seems to be independent of any underlying autoimmune hemolytic processes. It may reduce inflammation in the fibrotic marrow of MF patients, a notion which is supported by its simultaneous positive effect on platelet counts, but which has by no means been established. Hernandez-Boluda et al performed a retrospective study of prednisone among 30 patients with MF-related anemia.^40^ Starting dose was 0.5 to 1 mg/kg daily, with taper to minimum effective dose upon response. Standard IWG-MRT/ELN response criteria were used. Forty percent of patients achieved response at a median time to response of 1.1 months and median duration of response of 12.3 months. Fifty-five (6/11) percent of RBC transfusion-independent patients and 32% (6/19) of RBC transfusion-dependent patients demonstrated response. Responders demonstrated longer median survival (5.0 vs 1.5 years; *P* = 0.002). Common adverse events included hyperglycemia, cushingoid changes, and psychiatric disturbances. Prednisone may thus be considered in cases of refractory MF-related anemia without contraindication to corticosteroids, although its use is based on just 1 small retrospective study. Response is generally seen within 3 months and it should be tapered and discontinued if none is observed within that time. Like danazol, prednisone may also aid concomitant thrombocytopenia.

### Splenectomy

Sequestration and destruction of circulating RBCs is a major contributor to anemia among some patients with MF and splenomegaly.^14^ Ruxolitnib has done much to reduce the burden of splenomegaly among MF patients thus decreasing the need for, and incidence of, splenectomy in MF.^41^ Nevertheless, splenectomy remains a therapeutic option among patients with splenomegaly intolerant of or unresponsive to JAK inhibition. It does however remain a measure of last resort given its attendant morbidity and mortality as well as its rates of efficacy comparable to less invasive approaches. Tefferi et al reported a retrospective series of 223 MF patients from a single institution treated with splenectomy over a 20-year period.^42^ Among the 101 patients who had splenectomy for the primary indication of anemia, 37.6% demonstrated a response defined as achieving RBC transfusion-independence or an Hb level stably above 10 mg/dL. Of the 75 RBC transfusion-dependent patients, 30% achieved RBC transfusion-independence by 6 months postoperatively, and 23% maintained this benefit to the conclusion of study follow-up. Patients demonstrated a high incidence of perioperative mortality (9%) and morbidity (30.5%) across all indications with the most common complications including bleeding, infection, and thrombosis. An additional 16% of patients developed postsplenectomy hepatomegaly; however, few progressed to liver failure. Thus, splenectomy seems to offer response rates comparable to other established treatments for anemia in MF (with a somewhat greater degree of benefit among RBC transfusion-dependent patients) at the cost of significantly greater short-term morbidity and mortality. It thus generally remains a treatment of last resort.

### Immunomodulators

Immunomodulators have demonstrated mixed results at best with regard to MF-related anemia. The earliest such agent, thalidomide, was limited by tolerability, and while the latest iteration, pomalidomide, is significantly better tolerated its efficacy appears uncertain. In a phase 2 dose escalation trial of thalidomide in 63 MF patients conducted by Marchetti et al 39% of the 18 enrolled RBC transfusion-dependent patients were able to achieve transfusion-independence, an impressive response in this often intractable subgroup.^43^ However, 51% of all patients dropped out by 6 months due to toxicity (the most common adverse events being fatigue, sedation, constipation, and neuropathy). This was in spite of the low starting dose of 50 mg daily, and low median tolerable dose of 100 mg daily. In an attempt to improve tolerability, trials have combined thalidomide with prednisone. Mesa et al conducted a phase 2 trial of low-dose thalidomide (50 mg/day) combined with a 3-month prednisone taper in 21 MF patients.^44^ Ninety-five percent of patients completed 3 months of treatment. Four of the 10 RBC transfusion-dependent patients achieved transfusion-independence, and Hb increased by an average of 2.1 g/dL among transfusion-independent patients. However, it is unclear what proportion of this benefit was attributable to prednisone given its known independent efficacy in MF-related anemia.^40^ In order to help better clarify the efficacy and tolerability of thalidomide, Abgrall et al conducted a randomized controlled trial (RCT) of thalidomide 400 mg/day among 52 MF patients.^45^ Over half of the thalidomide group discontinued within 4 months and there was no difference in terms of anemia response between the thalidomide and placebo groups.

The thalidomide derivative, lenalidomide, has shown anemia-specific response rates of approximately 20% as monotherapy in phase 2 studies, but like its predecessor it has been limited by tolerability.^46,47^ The addition of prednisone was able to improve response rates to 30% in 1 trial; however, tolerability remains a major barrier even with adjunctive steroids, with the largest trial of lenalidomide/prednisone demonstrating an 88% incidence of grade 3 or 4 hematologic toxicity.^47^ Phase 1 and 2 studies of the third generation immunomodulator pomalidomide seemed to indicate response rates similar to those of its predecessor with the benefit of a substantially improved tolerability.^48–50^ However, an RCT of pomalidomide (RESUME trial) in 229 MF patients demonstrated no difference compared to placebo with respect to the primary outcome of RBC transfusion-independence (although it did demonstrate a significant platelet response among thrombocytopenic patients which was independent of anemia response; 22% vs 12% in placebo, *P* = 0.006).^51^ Although, initial reports from phase 2 testing of pomalidomide suggested differential effect in patient subsets including those harboring *JAK2V617F* and palpable splenomegaly <10 cm, such association with anemia response was not reproducible in multivariable analyses in the RESUME trial.^51,52^ Thus, as a group, immunomodulators have demonstrated only modest benefit for MF induced anemia, with thalidomide and lenalidomide exhibiting prohibitive tolerability, and thalidomide and pomalidomide failing to demonstrate significant efficacy in phase 3 trials.

## Emerging therapies

### Novel JAK Inhibitors

Given the vital role of the JAK-STAT pathway in erythropoietin mediated signaling it is not surprising that anemia is a prominent toxicity of the archetypal JAK inhibitor ruxolitinib.^53^ Thus, the finding that some among the second generation JAK inhibitors seem to relieve anemia has been quite unanticipated. Momelotinib (Gilead Sciences, Foster City, California, United States) is a JAK1/2 inhibitor which, unlike ruxolitinib, is associated with an anemia response in MF patients.^54^ The mechanism whereby momelotinib palliates MF-related anemia remains uncertain; however, it has been posited to inhibit ACVR1/ALK2 mediated hepatic production of hepcidin, thus prompting mobilization of storage iron and promoting erythropoiesis.^54,55^ Interim analysis of a phase 1/2 study investigating momelotinib in MF demonstrated an expected spleen response and, somewhat unexpectedly, a vigorous anemia response.^56^ An impressive 49 of 72 (68%) RBC transfusion-dependent patients achieved transfusion-independence at 12 weeks. Data on 100 patients from the above study enrolled at a single center were followed up for a median of 3.2 years with similarly encouraging findings including the achievement of RBC transfusion-independence in 51% of transfusion-dependent patients, and anemia response in 44% of all patients.^57^ Momelotinib also demonstrated effective palliation of splenomegaly and constitutional symptoms. Grade 3 or 4 thrombocytopenia was observed in 34% of patients. However, the most troubling toxicity was treatment emergent peripheral neuropathy, which although restricted to grades 1 and 2, occurred in 47% of patients and was often irreversible. A separate phase 1/2 trial of twice-daily momelotinib enrolling 61 subjects with MF demonstrated similar anemia response (45%) and similar rates of thrombocytopenia and neuropathy.^58^

Most recently, momelotinib has been directly compared with ruxolitinib in an RCT among 432 JAK inhibitor-naive MF patients (SIMPLIFY-1, NCT01969838).^59^ SIMPLIFY-1 achieved its prespecified primary endpoint of noninferiority to ruxolitinib for splenic response, however, failed to achieve non-inferiority in the key secondary endpoint of total symptom score (TSS). Momelotinib did outperform ruxolitib in reducing rates of transfusion-dependence (66.5% vs 49.3%, *P* < 0.001); however, the surprisingly high transfusion response rate in the ruxolitinib group may be cause for skepticism. Peripheral neuropathy rates with Momelotinib were lower compared with previous studies with similar incidence of neuropathy in the 2 groups (10% vs 9%). No patients discontinued due to neuropathy. A concurrent phase 3 trial, SIMPLIFY-2 (NCT02101268) has sought to compare momelotinib with best available therapy (BAT) including ruxolitinib as second-line treatments in MF patients previously treated with JAK inhibition.^60^ Although momelotinib failed to achieve the predefined threshold for spleen response, treatment was associated with improvement in disease-related symptoms and transfusion-independence (43.3% vs 21.2%, *P* < 0.001). Eleven patients (11%) in the momelotinib arm developed peripheral neuropathy, 3 of whom discontinued treatment. Although phase 3 data were disappointing with respect to spleen response, momelotinib would potentially have a role as a second-line agent for MF patients intolerant of ruxolitinib due to anemia, or a first-line agent for MF patients with prominent anemia would be unlikely to tolerate ruxolitinib initiation. However, the frequency of irreversible neuropathy remains troubling and will have to be further elucidated.

Pacritinib (CTI Biopharma, Seattle, Washington, United States) is a novel multikinase (including JAK2) inhibitor which, like momelotinib, has demonstrated promise in the alleviation of anemia. The mechanism whereby pacritinib avoids myelopsuppression remains uncertain; however, it may involve reduction of hematopoietic inhibitory cytokines via suppression of interleukin-1 receptor-associated kinase 1 (IRAK-1) or colony-stimulating factor 1 receptor (CSFR1).^61^ PERSIST-1, a phase 3 study of pacritinib versus BAT excluding ruxolitinib among patients with MF irrespective of baseline platelet count demonstrated pacritinib's superiority with respect to spleen volume reduction (SVR) (19.1% vs 4.7%, *P* = 0.0003) and TSS (25% vs 5.9%, *P* = 0.0001).^62^ Although the total number of transfusion-dependent patients was small, pacritinib therapy did result in significant RBC transfusion-independence. Twenty-six percent (9/36) of RBC transfusion-dependent patients who received pacritinib attained transfusion-independence versus 0% (0/16) of BAT patients (*P* = 0.043). This was followed be the PERSIST-2 study (NCT02055781), an RCT comparing pacritinib (2 arms: 200 mg twice daily and 400 mg once daily) and BAT including ruxolitinib in MF patients with baseline platelet count <100 × 10^9^/L.^63^ Although the combined pacritinib dose cohorts failed to demonstrate superiority over BAT with respect to SVR and TSS, the 200 mg twice daily cohort was statistically significant with 22% of those who received pacritinib 200 mg twice daily achieving SVR ≥35% (BAT = 3%, *P* = 0.001) and 32% achieving TSS reduction ≥50% (BAT = 14%, *P* = 0.011). Additionally, more pacritinib treated patients demonstrated reduction in transfusion-dependence (defined as a ≥50% reduction in average transfusions/month for 3 months relative to baseline), 15/72 (21%) of those receiving pacritinib versus 3/35 (9%) of those receiving BAT. Pacritinib was generally well tolerated; however, initial concerns surrounding excess deaths and cardiac and hemorrhagic events from interim analysis of the PERSIST program prompted the Food and Drug Administration to issue a full clinical hold on August 2, 2016. This hold has since been lifted (May 1, 2017) and a phase 2 dose exploration study in MF patients who have failed to respond to ruxolitinib has been initiated.^64^ While the anemia-specific response of pacritinib does not seem to be as impressive as that of momelotinib (acknowledging the difficulty of comparing across studies), pacritinib may offer another alternative to ruxolitinib for MF patients in whom JAK inhibition had previously been limited by baseline or treatment emergent anemia and/or thrombocytopenia.

INCB039110 (Itacitinib, Incyte Corporation, Wilmington, Delaware, United States) is a selective JAK 1 inhibitor presently in clinical development, which appears to have an anemia palliating effect. Results of a phase 2 open label trial (NCT01633372) of INCB039110 among intermediate and high-risk MF patients demonstrated clinically meaningful symptom relief, modest SVR, and impressive anemia response.^65^ Treatment with INCB039110 was not associated with induction of RBC transfusion-dependence among previously transfusion-independent patients. Only 3 of 48 (6%) patients who did not require RBC transfusions preceding treatment demonstrated significant transfusion requirement following initiation of treatment. More impressive were the responses among patients who had required RBC transfusions prior to study initiation. Of 39 such patients (who had received a median of 4 RBC units in the 12-week prestudy period) 6 did not require RBC transfusions during the treatment period, and 21 (53.8%) achieved a ≥50% reduction in RBC units transfused. INCB039110 was generally well tolerated although infections were common (44.8%), including upper respiratory tract infections in 19.5% of patients. However, most infections were mild or moderate, and only 4 grade 2 cases were felt to be treatment-related. Ideally, INCB039110, like pacritinib and momelotinib, would be able to extend the therapeutic benefits of JAK inhibition to MF patients who were previously not candidates due to anemia and possibly relieve RBC transfusion-dependence among a subset of patients.

## Anti-fibrotic agents

Progressive fibrosis of medullary hematopoietic tissue is central to the pathophysiology of anemia in MF.^6^ The development of agents capable of inhibiting or reversing this process represents a potential boon for MF patients struggling with intractable anemia. Numerous cytokines, signaling pathways, and stromal factors have been implicated in the perpetuation of the progressive bone marrow fibrosis characteristic of MF.^66^ Similarly, a number of endogenous factors have been identified which act to oppose fibrosis. One such factor, pentraxin-2 (serum amyloid P), is a circulating acute phase protein, which homes to sites of tissue damage and induces macrophage differentiation to prevent and reverse fibrosis.^67^ PRM-151 (Promedior), a recombinant form of pentraxin-2, has demonstrated anti-fibrotic activity in preclinical models of various fibrotic diseases.^68^ A multicenter phase 2 trial (NCT01981850) was conducted investigating the utility of PRM-151 at 2 dose levels (with and without ruxolitinib co-treatment) among 27 patients with intermediate- or high-risk MF.^69^ The primary endpoint was overall response by IWG-MRT/ELN criteria and/or decrease in bone marrow fibrosis by ≥1 grade. Nine of 26 evaluable (35%) patients demonstrated response with respect to the primary endpoint, and 6 of these 9 demonstrated response with respect to bone marrow fibrosis. Those patients who derived clinical benefit on study, 13 in total, were then allowed to continue PRM-151 beyond study conclusion, and the findings among this cohort following 72 weeks of therapy have been reported.^70^ Fifty-four percent of these patients demonstrated bone marrow morphologic response and significant response with respect to anemia was observed as well. Among 5 patients with a baseline Hb <10 g/dL median Hb increased by 24%, and among 5 patients with baseline transfusion-dependence, 3 achieved RBC transfusion-independence. PRM-151 was well tolerated with the most common adverse events being mild fatigue, nausea, and fever.

## Transforming growth factor -β ligand traps

Upregulation and dysregulation of proinflammatory cytokines is central to the pathogenesis of bone marrow fibrosis and failure of erythropoiesis that characterize MF.^15^ Among these oversecreted cytokines is transforming growth factor (TGF)-β which has been shown to stimulate collagen formation by marrow fibroblasts and facilitate the formation of mature collagen.^71,72^ In addition, TGF-β mediates aberrant stromal signaling which may inhibit or arrest terminal erythroid differentiation during erythropoiesis.^73^ TGF-β is thus a rational target for both the inhibition of pathological fibrosis and promotion of erythropoiesis. Sotatercept (ACE-011, Celgene) is a first-in-class activin receptor type IIA (ActRIIA) TGF-β ligand trap consisting of the extracellular domain of ActIIRA linked to the human IgG1 Fc domain.^74^ Sotatercept has shown efficacy in the treatment of anemia and reduction of RBC transfusion burden among patients with low and intermediate-risk MDS.^75^ The interim results of a phase 2 study (NCT01712308) show promising findings in MF-related anemia as well.^76^ All 18 patients enrolled were transfusion-dependent and responses, including transfusion-independence, were noted in 5 (36%) of 14 evaluable patients. The only adverse events attributable to sotatercept included grade 3 hypertension leading to discontinuation, and grade 1 myalgia and bone pain. A separate phase 2 study (NCT01712308) investigating the combination of sotatercept with ruxolitinib is presently ongoing. Luspatercept (Celgene) is a similar recombinant fusion protein which binds and traps ligands of the TGF-β superfamily.^77^ Trials are presently ongoing investigating the use of luspatercept in the treatment of anemia related to MDS (NCT02631070) and beta thalassemia (NCT02604433), and a phase 2 trial of MF subjects with anemia is to initiate (NCT03194542).

## Conclusions

The advent of ruxolitinib has done much to help palliate the splenomegly and constitutional symptoms which trouble MF patients.^78^ However, it has not been able to address the burden of anemia and, in fact, has further perpetuated it via its myelosuppressive properties.^17,18^ Anemia remains a stubborn challenge in the management of MF patients as it depresses quality of life, portends poor outcomes, and restricts access to JAK inhibition.^9^ Currently available treatments for MF-related anemia are inadequate with response rates reaching 30 to 40% at best among RBC transfusion-independent patients and worse among those who are transfusion-dependent, and often lacking durability.^39^ The benefit of ESAs is restricted to those patients with low endogenous S-Epo levels and even among these patients less than half respond and less than half of responders maintain response for over a year.^31–34^ The best option for those who are not candidates for or fail to respond to ESAs seems to be danazol.^38,39^ Although this agent has demonstrated the greatest efficacy among established treatments, it is able to elicit response in less than half of RBC transfusion-independent patients, and less than a quarter of RBC transfusion-dependent patients. Prednisone has demonstrated response rates approaching those of danazol; however, evidence for its use is based on a single limited retrospective study and is accompanied by a myriad of well-known toxicities when used long-term.^6,40^ Immunomodulators initially seemed promising; however, thalidomide and lenalidomide have been limited by impermissible toxicity, while pomalidomide failed to demonstrate significant efficacy in a pivotal phase 3 trial.^40,43–51^ Splenectomy offers some benefit as a last-line treatment; however, comes at the cost of significant short-term morbidity and mortality.^14,41,42^

Anemia thus remains a conspicuous unmet need in the management of MF. Fortunately, the forthcoming generation of novel therapies bodes significant promise. The second generation JAK inhibitor momelotinib has demonstrated unprecedented responses including the attainment of transfusion-independence in over 50% of transfusion-dependent patients in 2 separate early phase trials.^56,57^ However, irreversible peripheral neuropathy is a common and troubling toxicity, which will have to be overcome or risk mitigated. Pacritinib and INCB039110 offer somewhat more modest anemia responses than momelotenib; however, avoid significant neuropathy and may offer an excellent alternative to ruxolitinib among those patients with prohibitive anemia and/or thrombocytopenia.^63–65,79^ New classes of targeted agents including anti-fibrotics (PRM-151) and TGF-β Ligand Traps (sotatercept, luspatercept) offer rational therapies with demonstrated efficacy and minimal toxicity in early phase studies.^69,70,74,75^ As these agents proceed through further clinical trial evaluation and come under consideration for regulatory approval, they will need to prove substantial durable anemia response in a subset of MF patients that ideally can be identified upfront by a biomarker or clinical phenotype that would select for response (Tables 1–3).

**Table 1:**
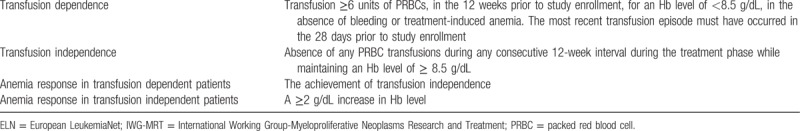
Useful Definitions From the (IWG-MRT)/ELN Response Criteria for Myelofibrosis^21^

**Table 2:**
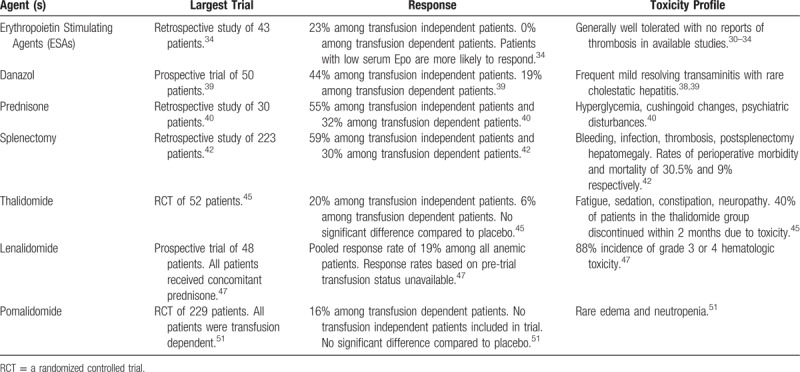
Established Treatments for Anemia in Myelofibrosis

**Table 3:**
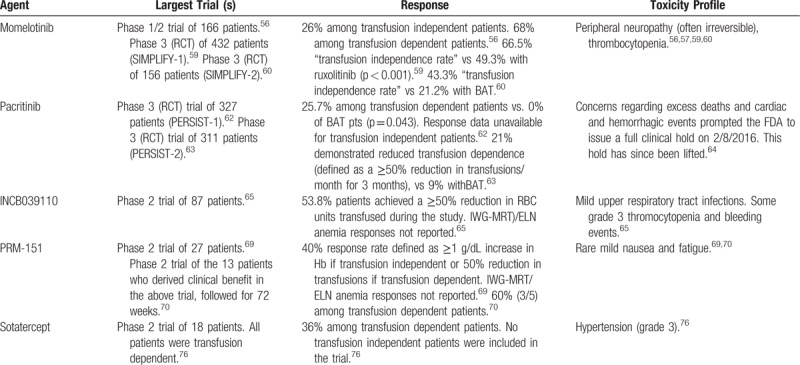
Emerging Treatments for Anemia in Myelofibrosis
